# Infantile Diarrhœa and Artificial Feeding

**Published:** 1896-03-07

**Authors:** 


### Infantile Diarrhœa and Artificial Feeding.

A recent investigation undertaken by Dr. James Niven, Medical Officer of Health for Manchester, in regard to the relation of infantile diarrhœa to methods of feeding on the one hand, and to condition of soil on the other, are full of interest as showing in what direction we must look for the prevention of this most fatal remedy.

It was long ago shown by Dr. Ballard that excessive prevalence of diarrhœa was associated with a certain temperature in the subsoil, and with insanitary conditions, especially such as imply the presence of filth in such form as to lead to fouling of the soil, the main facts being that the summer diarrhœa, which in some towns causes such a large infantile mortality, was a specific malady, and that the summer rise in this mortality followed very strictly the indications of the four-foot earth thermometer, the disease only commencing as an epidemic when the reading reached and remained above 56 degrees F., and the maximum mortality occurring in the week in which the maximum temperature was attained.

The interest of Dr. Niven's researches lies in the first place in the demonstration which they offer of the distinct relationship between infantile diarrhœa and artificial feeding. As the result of a large number of observations made by Dr. Tonkin and himself, and from inquiries instituted by his sanitary inspectors, he was able to arrive at an estimate of the proportion between infants of different ages who were (1) breast fed, (2) hand fed, (3) partially breast fed.

He then attempted to work out the influence of artificial feeding on the death-rate during the earlier months of life. The results are somewhat startling, and should make people hesitate to condemn their children to artificial feeding for any but the most urgent reasons. Thus it appears that the chance of death by summer diarrhœa under the age of one month in the infant fed wholly or in part by hand, to the chance of death of the breast-fed child at the same age, is at least 65 to 1; at two months, 75 to 1, &c.

In this calculation, however, a large number of children who are only partially hand-fed are included in the hand-fed category; but if we omit all these half-and-half cases, and compare only those which are entirely either one or the other, we find the following are the chances of the hand-fed child dying of diarrhœa, in comparison with those of the breast-fed child, at the various ages mentioned, viz.: Under one month, 88 to 1; one month, 95 to 1; two months, 72 to 1; three months, 58 to 1; four months, 123 to 1; and five months, 58 to 1.

Taken by itself, this is a striking commentary on the evils of artificial feeding as practised among the working classes. But a further inquiry into the relation between the partially hand-fed and those which are entirely either hand or breast fed, throws a very important light both on the mode in which artificial feeding is injurious and the mode in which the soil conditions, shown by Dr. Ballard to be related to the prevalence of diarrhœa, operate in producing their effect.

If the artificial food used for the infants of working-class people were merely unsuitable, and not in itself definitely injurious, while we might reasonably expect to find that the addition of it to a breast dietary would do a little harm, we certainly should not be justified in anticipating that in regard to diarrhœa mortality the mixed-fed infant should approximate so nearly as it does to the one brought up entirely on artificial food. The following table, which is a rearrangement of Dr. Niven's results, will illustrate the point we wish to emphasise:—

Chances of Death from Diarrhœa at Different Ages.

| | Under 1 month. | 1 month. | 2 months. | 3 months. | 4 months. | 5 months. |
|---|---|---|---|---|---|---|
| Hand fed to mixed fed | 1·9 to 1 | 1·5 to 1 | 4·5 to 1 | 2·6 to 1 | 3·2 to 1 | 4·5 to 1 |
| Hand fed to breast fed | 88 to 1 | 95 to 1 | 72 to 1 | 58 to 1 | 123 to 1 | 58 to 1 |

From this it is clear that the mixed-fed child allies itself with those which are brought up on artificial food so far as concerns the working-class population from which Dr. Niven's statistics are derived; in other words, that in towns in which summer diarrhœa prevails it is by way of the artificial food that the poison gains an entrance into its victim. Putting together, then, the proved relation of soil temperature, of insanitary conditions, and of artificial feeding in the production of infantile summer diarrhœa, it is hard to avoid the conclusion that it is in the storing and preparation of this food amid unclean surroundings, and in houses the air within which is largely drawn from a foul subsoil, that we have the immediate cause of this sad mortality.

Notwithstanding the warnings which have been given from time to time of the possibility of scorbutic conditions being set up by the use of sterilised food, the experience of certain hospitals and of many private practitioners has of late years tended to show that *where the surroundings are good* infants can be safely reared on artificial food. It is most important, however, to remember that this teaching cannot safely be made to apply to people who unfortunately have to live amid surroundings which are defective. The children may be reared, no doubt, but they run a fearful risk of being carried off by infantile diarrhœa whenever that is prevalent.

